# Combined targeting of TGF-β1 and integrin β3 impairs lymph node metastasis in a mouse model of non-small-cell lung cancer

**DOI:** 10.1186/1476-4598-13-112

**Published:** 2014-05-19

**Authors:** Elizabeth Salvo, Saray Garasa, Javier Dotor, Xabier Morales, Rafael Peláez, Peter Altevogt, Ana Rouzaut

**Affiliations:** 1 Oncology, CIMA, 31008 Pamplona, Spain; 2 DIGNA-Biotech, 31008 Pamplona, Spain; 3 Department of Translational Immunology, German Cancer Research Center, D015, Heidelberg D 69120, Germany; 4 Department of Biochemistry and Genetics, University of Navarra, 31080 Pamplona, Spain

**Keywords:** Integrin, TGF-β, Metastases, Lymph nodes, Lymphatic vessels

## Abstract

**Background:**

Transforming Growth Factor beta (TGF-β) acts as a tumor suppressor early in carcinogenesis but turns into tumor promoter in later disease stages. In fact, TGF-β is a known inducer of integrin expression by tumor cells which contributes to cancer metastatic spread and TGF-β inhibition has been shown to attenuate metastasis in mouse models. However, carcinoma cells often become refractory to TGF-β-mediated growth inhibition. Therefore identifying patients that may benefit from anti-TGF-β therapy requires careful selection.

**Methods:**

We performed in vitro analysis of the effects of exposure to TGF-β in NSCLC cell chemotaxis and adhesion to lymphatic endothelial cells. We also studied in an orthotopic model of NSCLC the incidence of metastases to the lymph nodes after inhibition of TGF-β signaling, β3 integrin expression or both.

**Results:**

We offer evidences of increased β3-integrin dependent NSCLC adhesion to lymphatic endothelium after TGF-β exposure. In vivo experiments show that targeting of TGF-β and β3 integrin significantly reduces the incidence of lymph node metastasis. Even more, blockade of β3 integrin expression in tumors that did not respond to TGF-β inhibition severely impaired the ability of the tumor to metastasize towards the lymph nodes.

**Conclusion:**

These findings suggest that lung cancer tumors refractory to TGF-β monotherapy can be effectively treated using dual therapy that combines the inhibition of tumor cell adhesion to lymphatic vessels with stromal TGF-β inhibition.

## Background

Lung cancer is a highly prevalent disease and is one of the leading causes of death worldwide. This neoplasia is usually detected in advanced stages and it has a 5-year survival rate of 20% [1]. Lung adenocarcinoma (AC) and lung squamous cell carcinoma (SCC) are the most common histological subtypes of lung cancer and they are generally smoking-related [2]. Tobacco contributes to the onset of lung carcinoma by inducing the expression of several cytokines including the molecule TGF-β, which is secreted by stromal fibroblasts [3]. TGF-β is a ubiquitous and pleiotropic cytokine that plays a dual role in cancer development. While it acts as a tumor suppressor in the early stages of the disease, at later stages of tumor development it contributes to malignant transformation through the activation of cell proliferation, metastasis and tumor angiogenesis [4,5]. Indeed, the production of TGF-β by tumor and stromal cells in response to radiotherapy and chemotherapy contributes to treatment resistance [6], and TGF-β inhibition in these cases improves treatment responses, particularly in models of solid carcinomas such as breast cancer [7].

The presence of lymph node metastasis is strongly associated with low survival rates in cancer patients [8,9], even in those diagnosed at early stages of the disease [10]. Tumor metastasis largely depends on the interaction between cancer cells and the tumor stroma. While host cells have tumor-suppressing capacities, malignancy induces several changes in the stroma (*e.g.,* tumor hypoxia) that eventually promote cell proliferation, invasion and metastasis [11]. Significantly, cytokines such as TGF-β play a key role in the transformation of the stroma during tumor development. Moreover, we have shown previously that TGF-β-induced factors are associated with worse overall prognosis in non-small-cell lung cancer (NSCLC) patients [12].

The lymphatic vessels constitute the main route by which solid carcinomas access the lymph nodes. Several studies have demonstrated that lymphangiogenesis is positively correlated with lymph node spread and adverse NSCLC prognoses [9]. Furthermore, both tumor and immune cells have been captured by electron microscopy in transit through channels formed in lymphatic endothelial cell (LEC) monolayers [13], although the molecular mechanisms by which tumor and immune cells enter lymphatic capillaries remain unknown. Lymphatic metastasis of NSCLCs may be facilitated by the specific morphological characteristics of the lymphatic endothelium. These vessels present an interrupted basal membrane [14] and their inter-endothelial junctional complexes are distributed in a dispersed button-like disposition [15]. Therefore, as it has been described for leucocytes, cell transit across these specific capillaries appears to be indolent [16]. Nevertheless, inflammation induces changes in the phenotype of the initial lymphatic vasculature [17] that elicit integrin-dependent mechanisms for an efficient recruitment of inflammatory cells [18,19].

As cancer is considered an inflammatory disease [20], it is important to determine whether integrins and their receptors also participate in tumor cell intravasation into the lymphatic vasculature. In fact, several studies have proposed an association between increased integrin expression in tumors and enhanced metastasis to the lymph nodes [21,22], and we previously demonstrated that hypoxia and nicotine promote the chemotaxis and adhesion of lung carcinoma cells to lymphatic endothelial cells [23,24]. In the present study, we examined the relationship between TGF-β exposure and tumor cell metastasis to the lymph nodes, and we sought to determine whether this relationship is mediated by integrin-dependent mechanisms.

## Materials and methods

### Cell culture and treatments

The human NSCLC cell lines H157, A549 and H1299, as well as cryopreserved primary Lung-Derived Human Lymphatic Microvascular Endothelial Cells (HMVEC-LLy, Lonza (Walkersville, MD, USA), were grown as described previously [12]. The cell lines were authenticated by PCR amplification of genomic DNA using specific primers for the specific CDKN2A mutation (c.205 G > T, in exon 2) and a KRAS mutation (c.34 G > C, in exon 2), and they were identified by the subsequent sequencing of the PCR products.

NSCLC cells were cultured in serum-free RPMI with 2 ng/ml human recombinant TGF-β (R&D Systems, Minneapolis, USA) for 24 h or 5 days. The medium was replaced and fresh cytokine was added every 48 h. For TGF-β blocking experiments, tumor cells were incubated with 10 mM of the TGF-βRI chemical inhibitor, SB431542 hydrate (Sigma-Aldrich, Steinheim, Germany), or 200 μg/ml of the TGF-β inhibitory peptide P144 (Polypetide Group, Strasbourg, France), 30 min before TGF-β treatment. Integrin αvβ3 blockade in H157 cells was achieved by adding 10 μg/ml of αvβ3-blocking antibody (MAB1976Z, Millipore, Billerica, MA, USA) 30 min before performing the assay. FAK was inhibited by incubation overnight with 1 μM PF-573228 (Sigma-Aldrich, Steinheim, Germany).

### Cell adhesion assays

Analysis of H157 cell adhesion to the lymphatic endothelium was performed as described previously [24]. Briefly, 3 × 10^4^ H157 cells were labeled for 20 min at 37°C with 10 μM calcein-AM (Sigma-Fluka, Steinheim, Germany), seeded on LEC monolayers and allowed to attach for 30 min at 37°C. Non-adherent cells were washed out and cell fluorescence was measured on a BMG Polar star Galaxy plate reader (Lab Technologies, Barcelona, Spain), using an excitation wavelength of 485 nm and a 520 nm emission filter.

### Cell transmigration assays

A total of 4 × 10^4^ LECs were seeded on 8 μm pore-size filters in modified Boyden chambers (BD Biosciences, San José, CA, USA) as described previously [19]. Next, 7 × 10^4^ H157 cells in 150 μl of serum-free RPMI medium were added and allowed to migrate for 24 h at 37°C towards the complete media added to the lower side of the filters. Transmigration efficiency was calculated as described previously [19].

The L1CAM and CD31 integrin receptors were blocked by pre-incubation of tumor cells or endothelial cells with blocking antibodies (20 μg/ml) for 1 h before carrying out the transmigration assays. The antibodies against human L1CAM (L1-9.3, directed against the L1CAM homotypic binding region, and L1-35.9, directed against the L1CAM RGD binding region) have been described previously [25]. The CD31 antibody was purchased from Sigma Aldrich (Steinheim, Germany).

### RNA isolation and PCR array

Total RNA was extracted with Trizol (Gibco, Carlsbad, CA, USA) according to the manufacturer’s instructions. For the PCR array, cDNA synthesis was carried out using 1 μg of total RNA and the RT^2^ First Strand Kit (SABiosciences, Qiagen Dusseldorf, Germany). Gene expression was profiled using the ECM and Adhesion Molecules RT^2^ Profiler™ PCR Array (SABiosciences, Qiagen Dusseldorf, Germany), according to the manufacturer’s instructions.

### Tumor cell transfection

H157 cells (1 × 10^6^ cells/ml) were transfected with 20 μg of a scrambled RNA or a HuSH^TM^ shRNA Plasmid Panels-29mer targeting integrin β3 (Origene, Rockville, MD, USA) in Opti-MEM medium (Invitrogen, Barcelona, Spain) using a Biorad Gene Pulsar I electroporator (Biorad, Berkeley, USA). Stable β3 integrin-silenced clones or cells expressing a non-specific scrambled RNA sequence were selected by culturing cells in the presence of 1.5 μg/ml puromycin-dihydrochloride antibiotic (Sigma-Aldrich, Steinheim, Germany). To generate GFP-expressing cells, H157 cells (2 × 10^5^) were transfected with 1 μg of the pEGFP-C1 plasmid (Clontech, Mountain-View, CA, USA) using FuGENE 6 Transfection Reagent (Roche, Barcelona, Spain), following the manufacturer’s instructions. Transfection efficiency was confirmed by flow cytometry and fluorescent microscopy, respectively.

### Western blot

Total cell protein extracts were prepared using RIPA buffer as described previously [12]. Membranes were blocked for 1 h with 10% non-fat milk or 5% BSA in TBS containing 0.1% Tween-20, and then incubated overnight at 4°C with the primary antibody at the dilutions recommended by the manufacturer.

The primary antibodies against FAK and phospho-FAK (Tyr397) were purchased from Cell Signaling (Danvers, MA, USA), and the anti-β-actin from Sigma-Aldrich (Steinheim, Germany). HRP-conjugated anti-rabbit IgG (Santa Cruz Biotechnology, Santa Cruz, CA, USA) was used as the secondary antibody. Blots were developed using Lumi-Light Plus Reagent (Roche, Barcelona, Spain), and the autoradiograms were scanned using a GS-800 calibrated densitometer and analyzed using Quantity One software (Biorad, Berkeley, CA,USA).

### Orthotopic mouse model of NSCLC

All protocols involving animal experiments were approved by the Experimentation Ethics Committee of the University of Navarra. Female athymic nude mice (5-6 weeks old) were purchased from Harlan Laboratories and GFP-H157 cells (1 × 10^6^) in PBS containing 10 μg of Matrigel (BD Biosciences, San José, CA, USA) were injected in a total volume of 20 μl into the left lung of these nude mice as described previously [26]. Each mouse was then injected intra-peritoneally with either vehicle (PBS) or 200 μg of the TGF-β inhibitor peptide P144 daily. Mice were sacrificed 28 days after treatment or upon exhibiting symptoms of cachexia. Primary tumors and brachial and axillary lymph nodes from both sides were extracted, fixed in Bouin solution and paraffin-embedded for histopathological analysis.

### Immunohistochemistry and confocal microscopy imaging

Endogenous peroxidase activity was quenched in formalin-fixed paraffin-embedded tissue sections (3 μm thick) and they were then exposed to microwaves. Non-specific binding was blocked by incubation for 30 min in 5% goat serum in TBS, before the sections were incubated overnight at 4°C with antibodies against GFP (1:1000, Abcam, Cambridge, UK) or β3 integrin (1:1000, Chemicon/Millipore, Billerica, MA, USA). The sections were then incubated for 30 min at room temperature with Envision polymer (Dako) to increase the signal intensity. Peroxidase activity was visualized with diaminobenzidine, and the sections were counterstained with hematoxylin and mounted in DPX mounting medium (BDH Chemical, Poole, UK). GFP staining was scored qualitatively and expressed as the proportion of positive cells (0–100%), as described previously [12].

Cells were seeded onto 35 mm glass-bottom culture dishes (MatTek Corporation, Ashland, MA, USA) for confocal microscopy (Ultraview ERS, PerkinElmer, Waltman, MA, USA) and the images from stacks (0.5 μm deep) were captured every 2 min over 2 h using a 63× water objective, and they were analyzed using Ultraview ERS (PerkinElmer, Waltman, MA, USA) and FIJI (Image J, Bethesda, MA, USA) software.

### Primary tumor growth analysis

Tumor growth was quantified using FIJI software (Image J) on microphotograph images obtained on a Zeiss Axio Imager M1 microscope (Carl Zeiss, AG, Oberkochen, Germany) from fixed samples. The methods and parameters used for micro-CT image acquisition and image reconstruction have been described elsewhere [27].

### Statistical analysis

Normally distributed data were analyzed using a Student’s *t*-test or ANOVA followed by post-hoc analyses. Data with a non-parametric distribution were analyzed using the Kruskal-Wallis and Mann–Whitney U-tests. Mouse survival was analyzed using the log-rank test. Differences were considered significant at p < 0.05. All analyses were performed using SPSS 15.0 or Graph Pad Prism 5 software.

## Results

### TGF-β exposure enhances H157 NSCLC cell adhesion and transmigration across lymphatic endothelial cell monolayers

To establish an in vitro system in which to study our hypothesis we first evaluated the response of three NSCLC cell lines (H1299, H157 and A549) to TGF-β by measuring SMAD2 phosphorylation and its inhibition by cell exposition to the specific inhibitor of the TGF-β receptor Type I kinase SB431542, or to P144, a TGF-β-binding inhibitory peptide obtained from the sequence of the human TGF-β receptor type III (beta-glycan). We observed that although both inhibitors specifically diminished phospho-SMAD signal, P144 inhibited SMAD2 phosphorylation to a lower extent (Additional file 1: Figure S1A). In our view, SB431552 inhibits more intensely SMAD2 phosphorylation because it specifically targets TGF-βRI kinase and therefore the subsequent phosphorylation of SMAD, while P144 is a short peptide derived from the sequence of the TGF-βRIII (beta-glycan) that binds to soluble TGF-β and blocks TGF-β signaling through all its possible receptors. To study the effect of TGF-β on cell dynamics we performed cell migration assays to analyze cell movements towards chemotactic factors. Cell migration was enhanced in NSCLC cells exposed to TGF-β (Additional file 1: Figure S1B and C). Based on these findings, we selected the H157 NSCLC cell line with which to model the TGF-β response of lung cancer cells.

**Figure 1:**
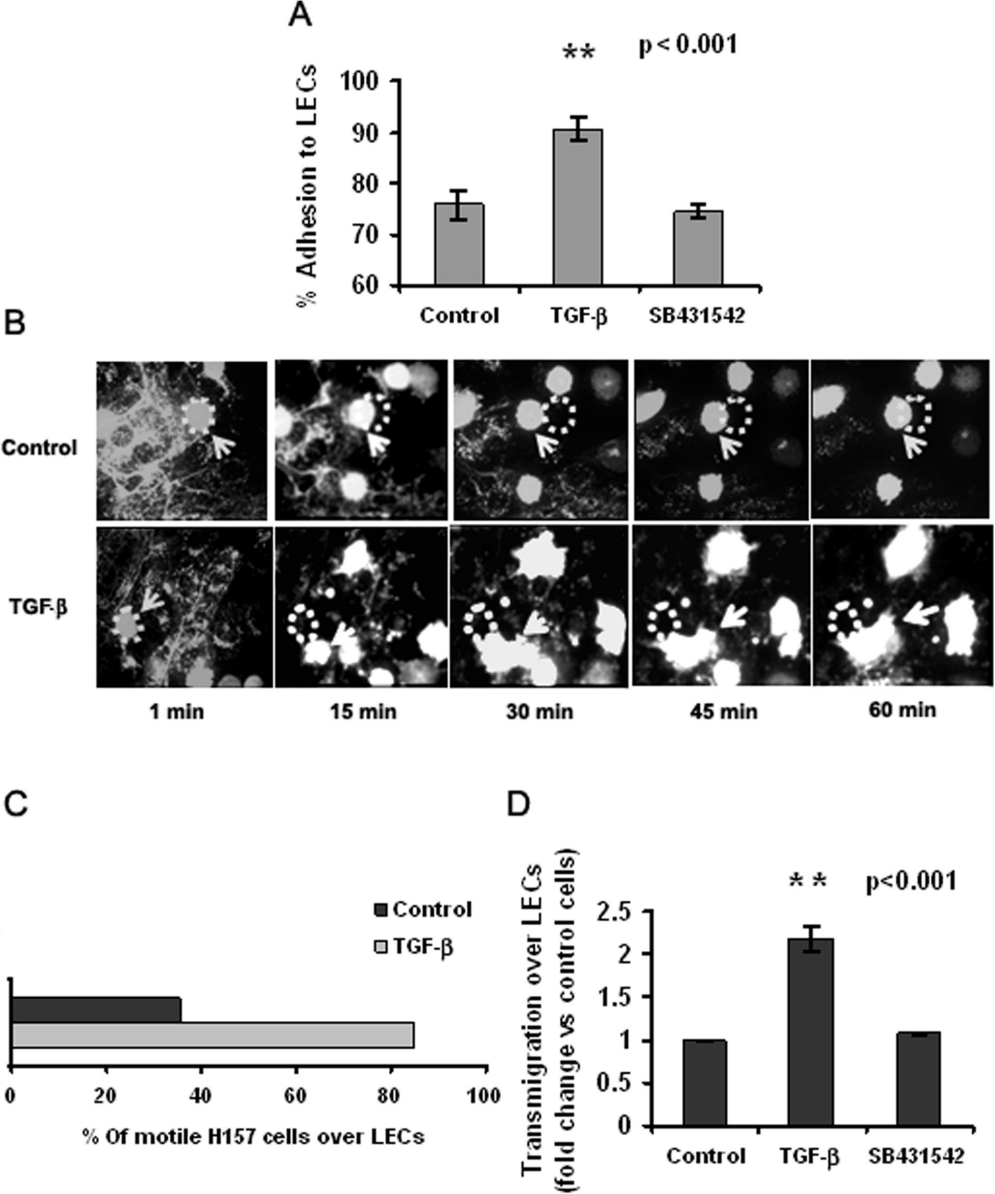
**TGF-β exposure enhances NSCLC cell adhesion and transmigration across lymphatic endothelial cells. (A)** Adhesion of H157 cells to LEC monolayers after treatment for 5 days with TGF-β (2 ng/ml), and in the presence or absence of the TGF-βRI inhibitor SB 431542 (**p < 0.001, Student’s *t*-test). **(B)** Time-lapse microphotographs of the movement of H157 NSCLC cells, treated as in A, across LEC monolayers (63× water objective). Dots indicate the initial position of the cell and arrows indicate the same cell in motion. **(C)** Quantification of the number of H157 cells in transit over LEC monolayers, expressed as a percentage of the total number of cells counted in a single XY plane. **(D)** Quantification of NSCLC cell transmigration across monolayers of primary human LECs in the presence or absence of TGF-β. Data are presented as fold-change with respect to untreated control H157 cells (**p < 0.001, Student’s *t-*test).

To determine whether TGF-β enhances NSCLC cell migration through lymphatic vessels, we studied H157 cell adhesion and transmigration across monolayers of primary human LECs. TGF-β treatment increased cell adherence to LEC monolayers and altered cell motility when measured by video microscopy (Figure 1A and B). Indeed, while only 30% of untreated cells moved on the endothelial surface (by blebbing), in the presence of TGF-β the number of motile cells multiply three fold and moved by emitting filopodia, indicative of integrin-mediated displacement [28] (Figure 1B and C). We also tested whether TGF-β treated cells traversed LEC monolayers at greater intensity in Boyden chambers assays. Results show that it was the case: cell transmigration across endothelial layers was increased more than two-fold in TGF-β-treated cells (Figure 1D). As expected, this increment was abrogated when cells were incubated with the TGF-βRI inhibitor SB431542, indicating that this effect is specific to the cytokine.

### Integrin mRNA expression is increased in TGF-β-treated cells

**Figure 2:**
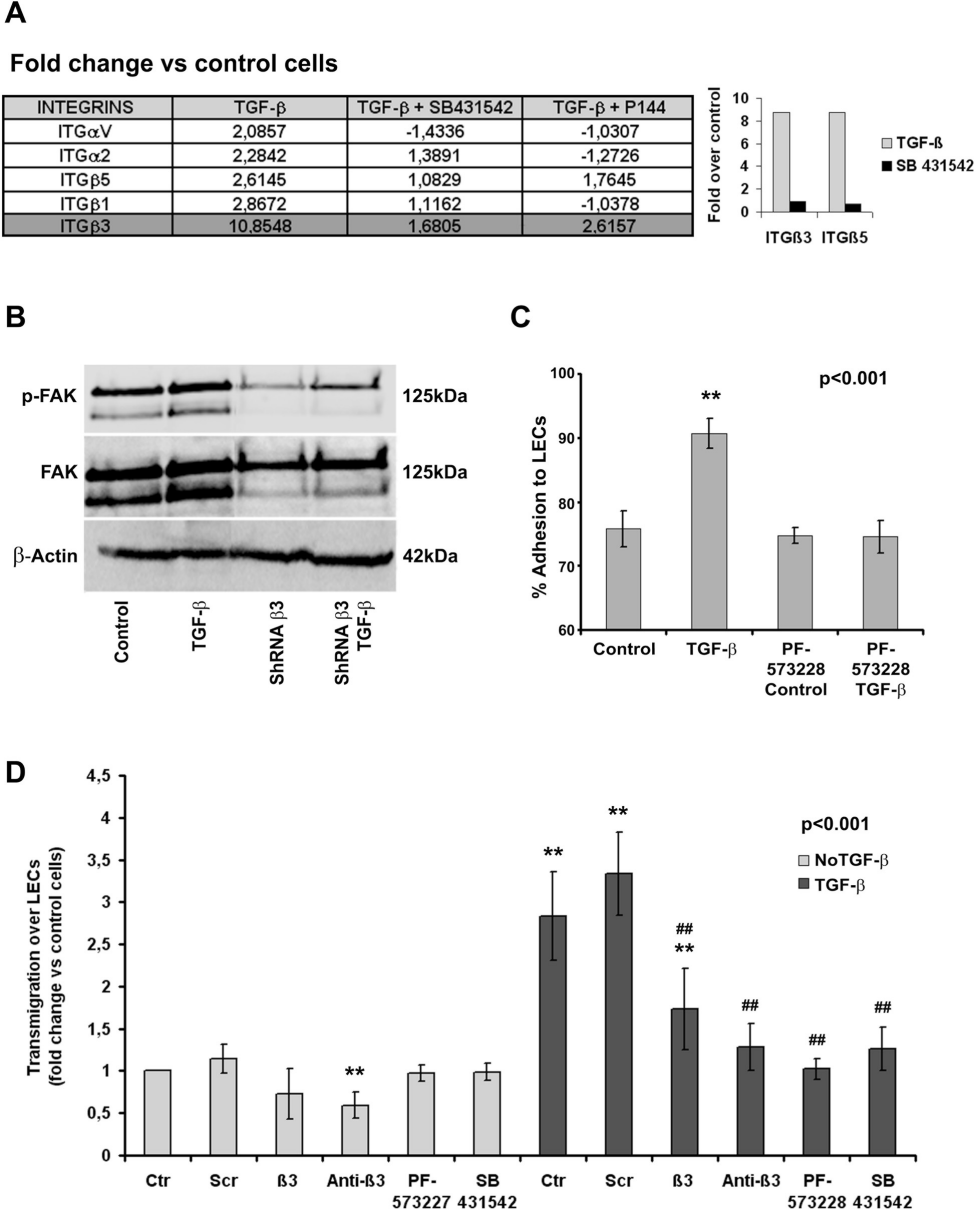
**TGF-β treatment induces integrin expression, FAK phosphorylation, β3 integrin-dependent adhesion and transmigration of H157 NSCLC cells across LEC monolayers. (A)** mRNA expression of several integrins in H157 cells following treatment with TGF-β and its inhibitors (fold-change with respect to untreated cells) and confirmation by real-time PCR of the differential expression of β3 and β5 integrins after exposure to TGF-β. **(B)** FAK phosphorylation after TGF-β treatment of β3 integrin-deficient (shRNAβ3) and β3 integrin-competent H157 NSCLC cells. **(C)** Adhesion of TGF-β-treated H157 cells to LEC monolayers in the presence or absence of the FAK inhibitor, PF-573228 (**p < 0.001, Student’s *t-*test). **(D)** Quantification of H157 cell transmigration across LEC monolayers in the presence of the TGF-βRI inhibitor SB431542, the FAK inhibitor PF-573228, a blocking mAb against β3 integrin (Anti-β3) and that of β3 integrin-deficient H157 clones (β3). Samples were pretreated with or without TGF-β as described in the Materials and methods (**p < 0,001 compared against non-treated cells, ^##^ p < 0,001 compared against TGFβ-treated cells, Mann–Whitney *U*-test).

To obtain a metastases-related mRNA signature specific to TGF-β-treated H157 NSCLC cells, we used the SABiosciences RT^2^ Profiler™ PCR Array that measures the expression of 94 genes related to adhesion molecules, proteases and extracellular matrix components. Interestingly enough, TGF-β induced increases in the expression of several integrins, such as α2, αv, β1 integrins and most prominently, β3 integrin (Figure 2A) as it has been described in other systems [29]. Besides, major changes in the expression of genes encoding extracellular matrix proteins were observed, including collagens type I, VII and XIV, fibronectin and laminin (Additional file 2: Figure S2). We also observed increased expression of MMPs, ADAMTS, TIMP and CTGF, among other genes. To control for the specificity of TGF-β induction we hybridized the arrays with samples treated with SB 431542 or with P144, a peptide inhibitor of TGF-β developed in-house [30]. Accordingly, the differential expression of 18 selected genes was confirmed by Real-Time PCR, including all the integrins detected (Additional file 2: Figure S2). Of interest, we observed that although the majority of the genes (26/31) responded to both inhibitors in the same sense (diminishing or incrementing gene expression), some differences in the intensities of the responses were detected. These variances can be due to their diverse targeting molecules: while P144 binds to TGF-β, SB431542 specifically inhibits the phosphorylation of one of its receptors namely TGF-βRI. In this sense, five genes presented completely opposite responses depending on the inhibitor used: MMP-10, MMP14, SPARC were induced after treatment with P144 and inhibited by SB431542. These results suggest the existence of TGF-β-dependent but TGF-βRI-independent inhibitory mechanisms involved in the regulation of their transcription. On the contrary E-Selectin and MMP3 expression was induced after treatment with SB431542 and inhibited as a result of P144 exposure. Thus, since SB431542 targets only one of the possible TGF-β-induced signaling pathways and P144 blights all the different pathways activated by this cytokine, we selected P144 for our experiments in order to target stromal TGF-β and inhibit all its effects at once.

### β3 integrin is required to mediate the TGF-β driven increases in cell transmigration across LECs

Based on the significant induction of integrin expression observed in our experimental conditions, we investigated the role of integrins in NSCLC adhesion to LECs. Exposure to TGF-β induced the phosphorylation of the focal adhesion kinase (FAK) in H157 cells, a kinase that mediates integrin activation in response to TGF-β treatment (Figure 2B). To confirm the participation of the integrin signaling pathway in cell adhesion to LEC monolayers, we performed adhesion experiments with H157 cells pretreated with PF-573228, a chemical inhibitor of FAK. After FAK inhibition, the number of cells that adhered to LECs decreased to levels observed in untreated cells (Figure 2C). Curiously, PF-573228 did not reduce tumor adhesion to LEC monolayers in control cells. These findings indicate that FAK activation occurs exclusively after exposure of H157 NSCLC cells to TGF-β and that it mediates cell adhesion to LEC monolayers.

**Figure 3:**
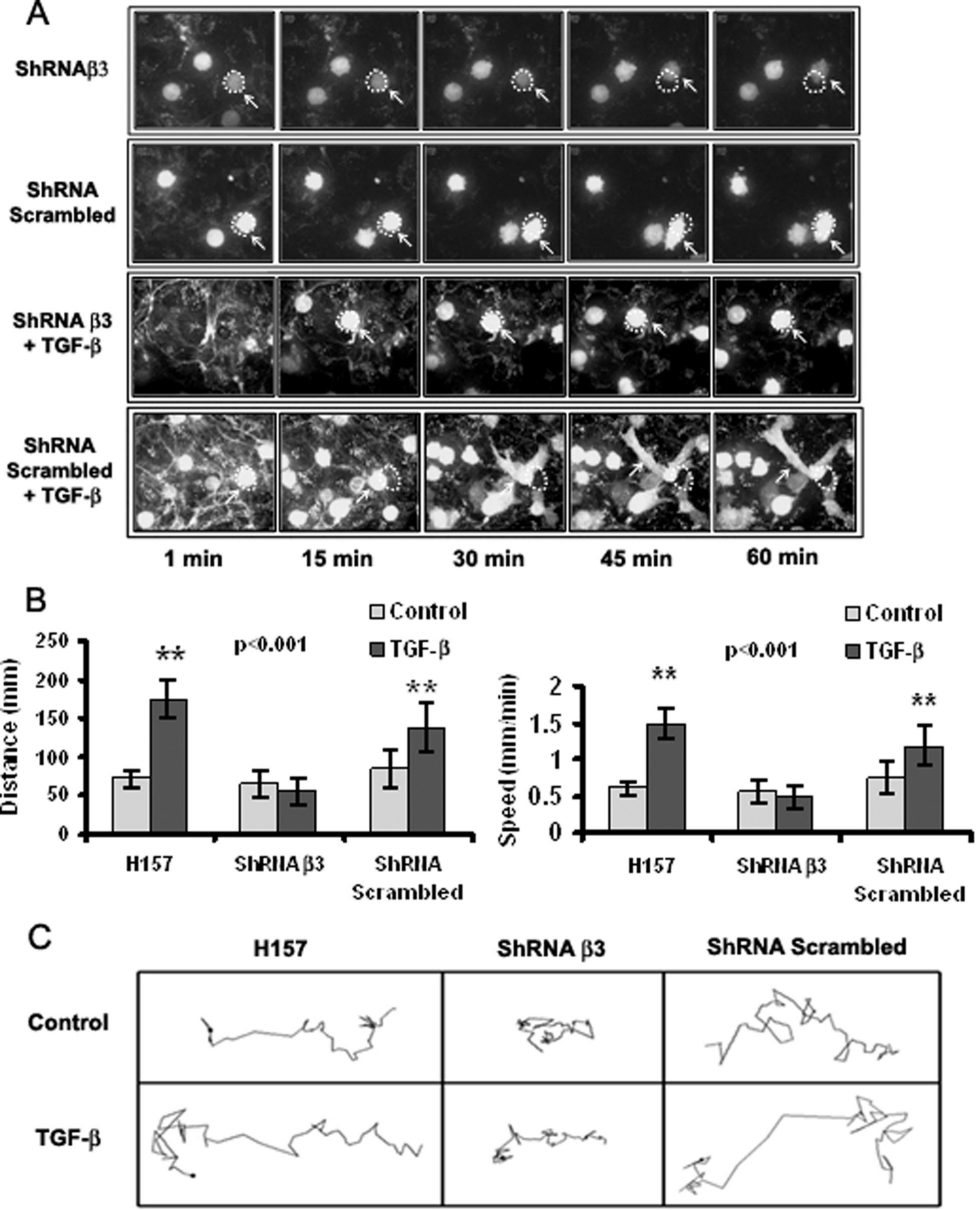
**Integrin β3 silencing in H157 cells significantly diminishes cell movement across LEC monolayers. (A)** Microphotographs showing the adhesion of TGF-β-pretreated H157 cells (integrin-competent and integrin-deficient) to LEC monolayers (63× water objective). **(B)** Average distance covered and speed of TGF-β-pretreated H157 cells (integrin-competent and integrin-deficient): ** p = 0.001, one-way ANOVA with Dunnett’s multiple comparison test. **(C)** Representation of cell directionality for one representative cell from each group. A minimum of 30 cells were analyzed per group.

To specifically demonstrate the participation of these pathways in tumor cell transmigration across LEC monolayers, we performed transmigration assays using cells treated with the TGFβ-RI kinase inhibitor SB431542, the FAK inhibitor PF-573228, or after the cells had been pre-treated with a blocking antibody against the β3 integrin. We also developed H157 clones that were stably transfected to express β3 integrin-specific shRNAs. As it is demonstrated in Figure 2D, inhibition of FAK or TGF-β signaling and of β3 integrin expression or functionality severely impairs the transmigration of TGF-β treated H157 cells. Importantly, these effects were not detected or were significantly smaller (*e.g.* treatment with β3 integrin blocking antibodies) in control cells. Therefore, TGF-β pre-treatment induces incremented cell transmigration across monolayers of lymphatic endothelial cells in a manner that is dependent on the activation of TGF-βRI and FAK signaling pathways and on the intervention of β3 integrin subunits.When we analyzed H157 cell dynamics on LEC monolayers by confocal video microscopy, we observed that β3 integrin expression was required for cells to move across LEC monolayers, to adopt a fibroblast-like morphology and to extrude filopodia (Figure 3). In fact, we found no differences in the average speed and distance covered between β3 integrin-silenced cells pretreated with TGF-β and untreated control cells. Together, these findings demonstrate that the TGF-β-dependent increases in tumor cell adhesion and transmigration across LEC monolayers are mediated by β3 integrin expression at the tumor cell surface.

**Figure 4:**
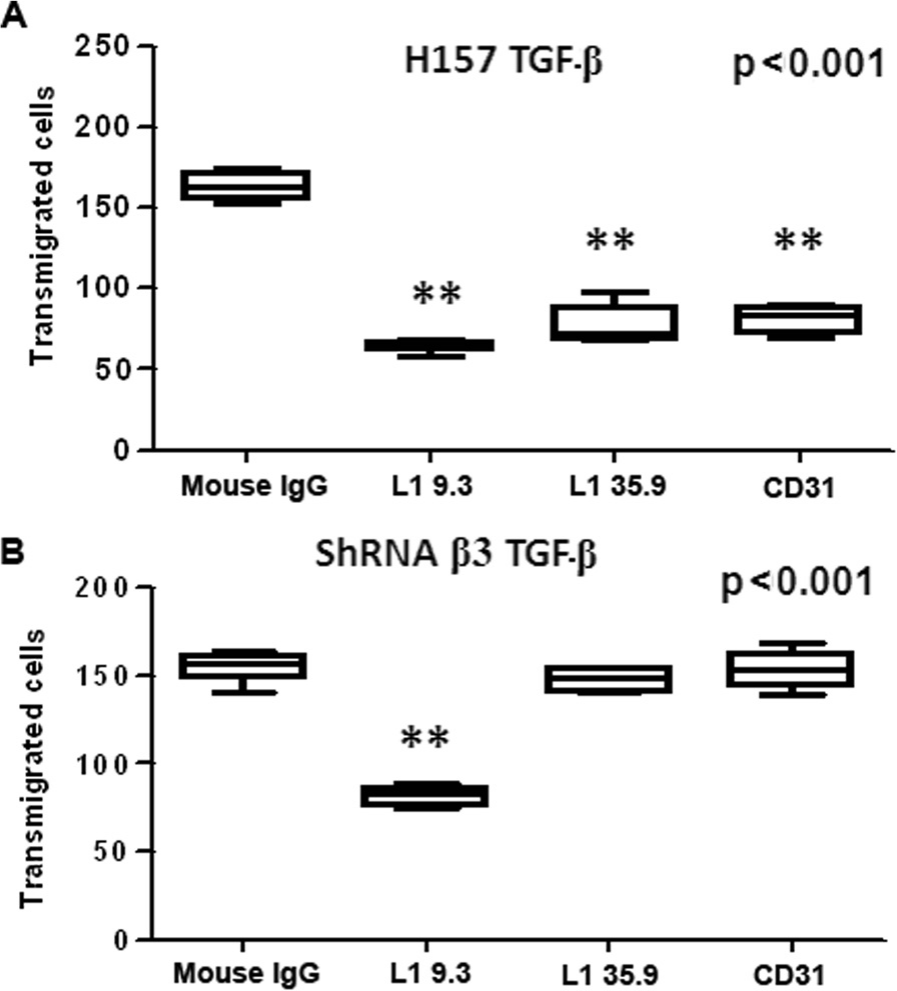
**H157 cell transmigration across endothelial monolayers is inhibited by blocking antibodies against the β3 integrin ligands L1CAM and CD31.** Quantification of the number of TGF-β-pretreated β3 integrin-competent **(A)** and β3 integrin-deficient **(B)** H157 cells transmigrating across LEC monolayers in the presence of blocking antibodies against the L1CAM RGD binding region (L1.35.9 mAb), the L1CAM homotypic binding region (L1 9.3 mAb) and CD31: **p = 0.001, one-way ANOVA with Dunnett’s multiple comparison test.

L1CAM and CD31 are β3 integrin ligands that are expressed on the surface of LECs (Additional file 3: Figure S3). L1CAM has been implicated in tumor metastasis and therapeutic antibodies that target this molecule block tumor growth in experimental models of ovarian and pancreatic cancer [31,32]. To investigate whether these receptors participate in the transmigration of H157 cells across LEC monolayers, we performed transmigration assays in the presence of blocking antibodies against the L1CAM RGD binding region (L1-35.9 mAb), the L1CAM homotypic binding region (L1-9.3 mAb) and CD31. All three blocking antibodies reduced the transmigration of TGF-β-treated H157 tumor cells across LECs by 50% with respect to the corresponding controls (Figure 4A). As L1CAM and CD31 can interact via homotypic contacts, we studied the effect of blocking these ligands on β3 integrin-dependent cell transmigration across LECs. As such, when we repeated the transmigration experiments with β3 integrin-silenced H157 cells, their adhesion to LECs was only reduced by the anti-L1-9.3 antibody that blocks L1CAM homotypic binding (Figure 4B). Hence, H157 cells appear to bind LEC via L1CAM homotypic and L1CAM/integrin β3 and CD31/integrin β3 heterotypic binding. Interestingly, when cells were simultaneously incubated with both L1CAM blocking antibodies prior to performing the adhesion experiments, the efficiency of blocking was unchanged and remained at 50% of the control levels (data not shown). These data suggest that binding of an L1CAM-blocking antibody impedes subsequent binding or the function of the other blocking antibody.

### TGF-β and integrin β3 expression influences cell survival and tumor growth in a mouse model of orthotopic lung cancer

**Figure 5:**
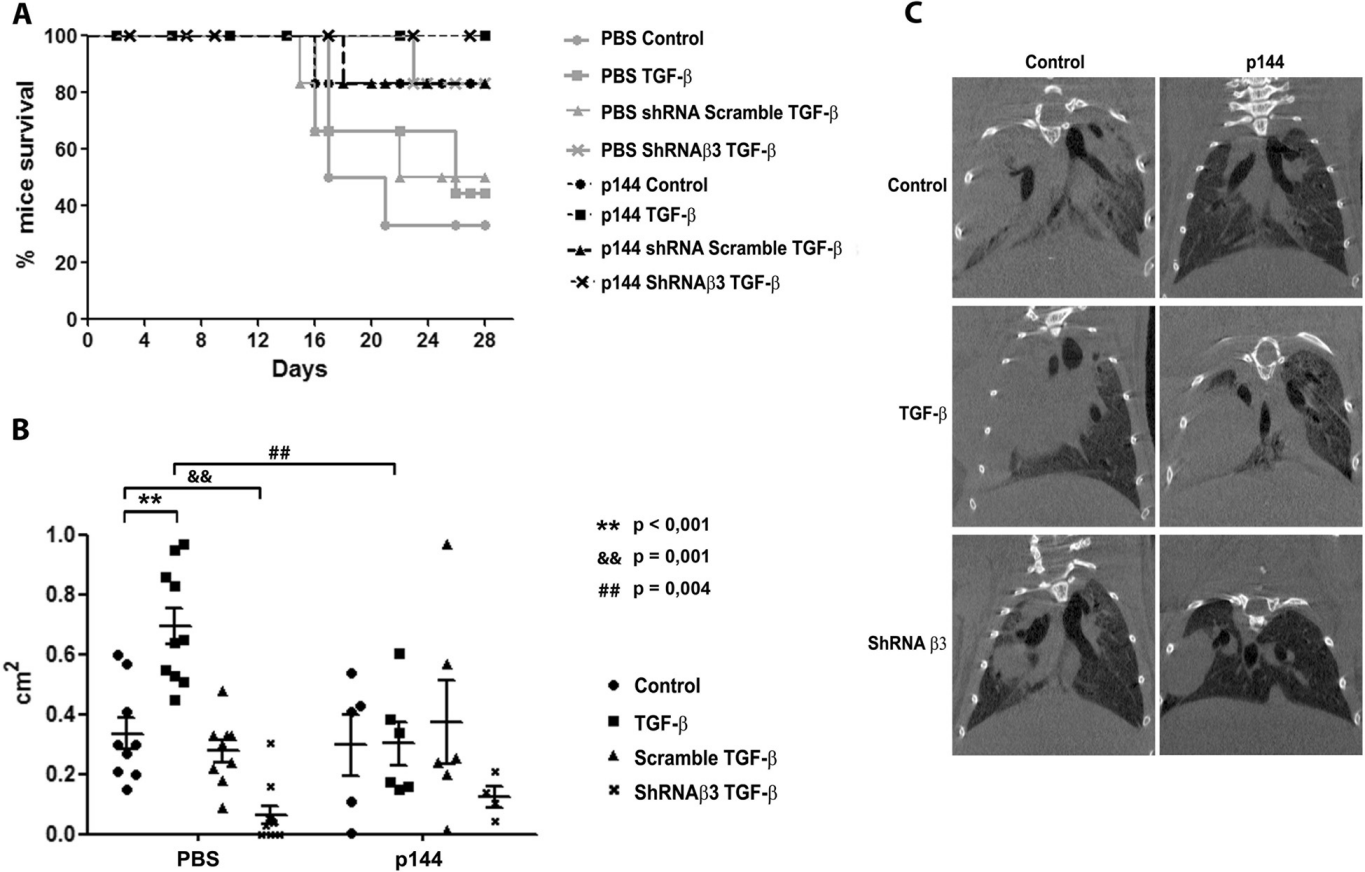
**Integrin β3 silencing and inhibition of stromal TGF-β improves survival and attenuates tumor cell growth in an orthotopic mouse model of lung carcinoma. (A)** Kaplan-Meier survival curves for mice injected with β3 integrin-deficient or β3 integrin-competent cells that were pretreated with or without TGF-β. Mice were injected intraperitoneally with 200 μg of the TGF-β inhibitor peptide P144 as described in Materials and methods. **(B)** Tumor size determined from microphotographs obtained from tumor sections and expressed in cm^2^ (maximum diameter). Image analyses were performed using fiji software. Data were analyzed using a one-way ANOVA followed by a Dunnett’s multiple comparison test (** p < 0.001: H157 TGF-β PBS vs H157 control PBS; ^§§^p = 0.001: shRNA β3 TGF-β PBS vs H157 control PBS; ^##^p = 0.004: H157 TGF-β PBS vs H157 TGF-β P144). **(C)** Micro-CT analyses of mouse lungs before sacrifice. A representative image from each group is shown.

To validate our in vitro findings in an in vivo setting, we developed an orthotopic model of lung cancer by directly injecting integrin β3-deficient or integrin β3-competent H157 cells into the lungs of immune-deficient mice, with or without TGF-β pretreatment. To study the importance of stromal-derived TGF-β, mice received daily intraperitoneal injections of the TGF-β inhibitor peptide P144 (200 μg), and survival was analyzed by Kaplan-Meier curves (Figure 5A). No significant differences in survival were observed between mice injected with H157 cells previously exposed to TGF-β or not. By contrast, the survival of mice injected with β3 integrin-silenced tumor cells was significantly higher, increasing from 30% to 80% that of the controls. In some cases mice injected with cells transfected with commercial non-specific shRNA showed mixed responses, even though these cells were successfully used in vitro. Indeed, further analysis of this RNA sequence revealed some similarity (60%) with the RNA sequences of bone morphogenic protein 2 (BMP2) and SMAD5, both of which are involved in TGF-β signaling, which may explain the source of these spurious results. Inhibiting stromal TGF-β by intraperitoneal administration of P144 increased the survival rates in all groups regardless of whether the cells injected were untreated or pretreated with TGF-β.

Tumor histology was analyzed after sacrificing the mice, revealing that H157 tumor cells pretreated with TGF-β formed larger tumors than untreated cells. Moreover, this growth was abrogated when mice were treated with the inhibitory peptide P144 (Figure 5B), while the smallest tumors were detected in animals injected with integrin β3-silenced cells (mean size = 0.06 cm^2^). These findings were supported by the results of micro-CT analyses of mice prior to sacrificing. In mice injected with integrin β3-silenced cells and treated with the TGF-β inhibitor peptide P144, tumor affected lung area was smaller than that observed in control samples (Figure 5C). Hence, the inhibition of cell adhesion through integrin silencing and/or the inhibition of stromal TGF-β limit tumor growth and favors survival in our experimental model.

### Concomitant TGF-β1 inhibition and integrin β3 silencing decreases lymph node metastasis in mice

**Figure 6:**
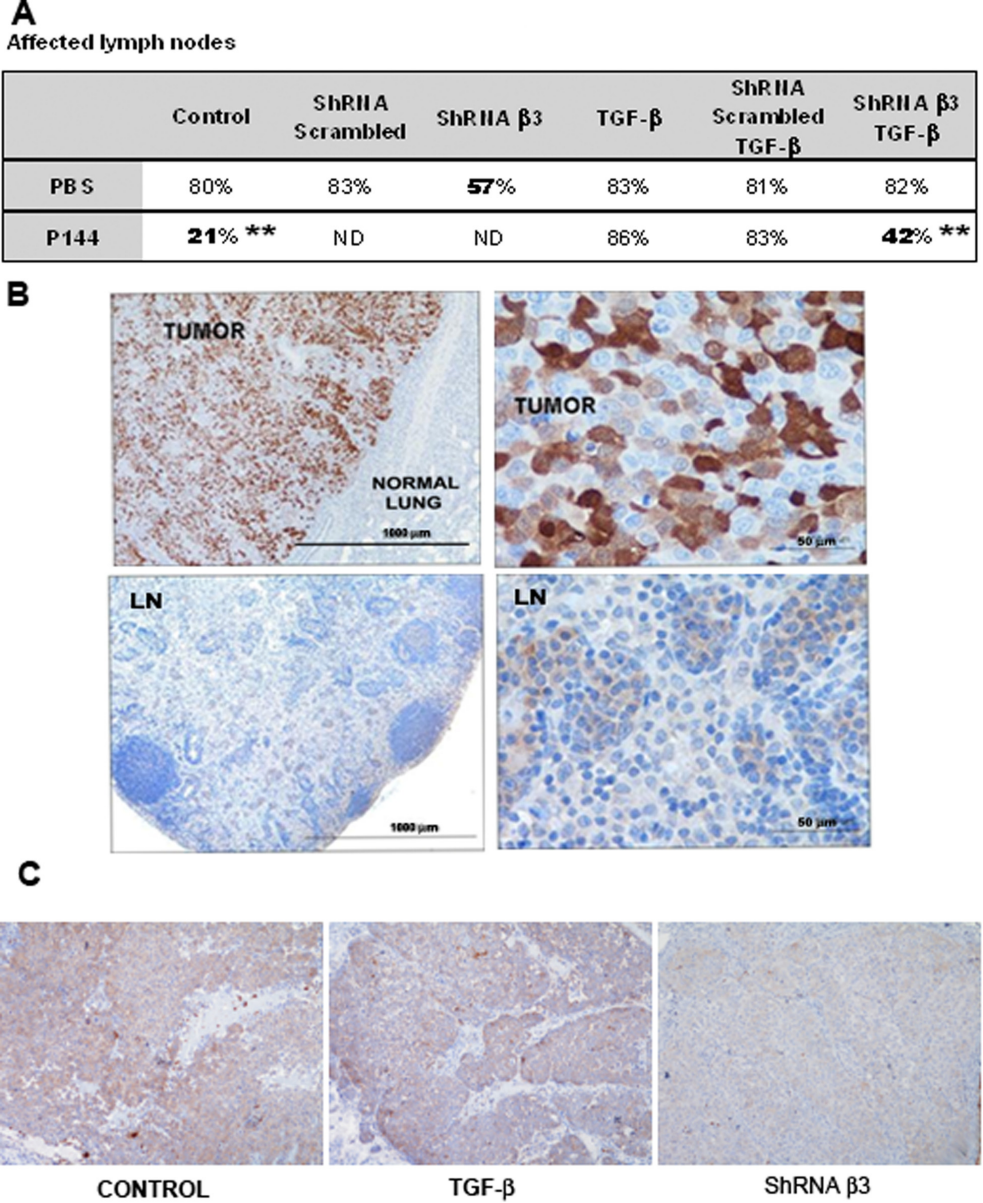
**Combined targeting of TGF-β and integrin β3 attenuates lymph node metastasis of H157 NSCLC cells in the lungs of immunodeficient mice. (A)** Percentage of affected lymph nodes (axillary and brachial) in the different experimental groups analyzed. Statistical analyses were performed by means of Mann–Whitney U- test. Significant values are depicted with double asterisks and correspond to p values of 0.009 for both comparisons **(B)** Representative microphotograph of GFP tumor detection in the histological samples obtained from lungs (upper row) and lymph nodes (lower row). **(C)** Immunohistochemical detection of integrin β3 in tumors obtained from mice.

Since our in vitro results suggested the participation of β3 integrin in H157 cell transmigration across LECs, we quantified the percentage of lymph nodes affected by tumor cells in each of the experimental groups (Figure 6). TGF-β pretreatment of H157 cells had no effect on their ability to form metastatic foci in lymph nodes. In contrast, in mice injected with untreated cells, the inhibition of stromal TGF-β by intraperitoneal injection of P144 resulted in an important diminution of the incidence of metastasis to the lymph nodes from 80% to 21% (p = 0.009) with respect to control animals. Furthermore, mice injected with H157 cells in which β3 integrin had been silenced displayed less lymph node affectation than those injected with β3 integrin-competent cells (57% vs. 80%) (p = 0.24).

We observed significant variation in the results when mice were injected with H157 cells that had been pretreated with TGF-β in vitro. In this case, lymph node affectation did not differ between mice that received β3 integrin-competent and β3 integrin-deficient cells, with rates of ~80% observed in both groups of mice. This suggests that a compensatory mechanism is triggered in H157 cells after TGF-β exposure that enables them to overcome the lack of β3 integrin and promote cell migration towards the lymph nodes. The inhibition of stromal TGF-β by intraperitoneal injection of P144 also failed to prevent metastasis to the lymph nodes in mice injected with β3 integrin-competent H157 cells that were pretreated with TGF-β. Thus, TGF-β pretreatment allowed tumors to overcome the specific silencing of integrin β3 expression or the inhibition of TGF-β in the tumor stroma. Importantly, when we injected β3 integrin-deficient H157 cells that had been pretreated with TGF-β in mice that were subsequently treated with P144, the incidence of lymph node affectation dropped from 80% to 42% (p = 0.009). These findings indicate that concurrent targeting of integrin β3 and TGF-β signaling significantly attenuates the incidence of lymph node metastases in cells that have evolved towards more aggressive phenotypes due to TGF-β exposure.

## Discussion

The induction of angiogenesis, invasion and metastasis by TGF-β in advanced stages of cancer has been well demonstrated [29]. Accordingly, the inhibition of TGF-β-mediated signaling has aroused great interest in the scientific community as a potential therapeutic approach to cancer treatment. Small molecule inhibitors such as the TGF-βRI inhibitors LY2157299, SB-50124 and SM16, monoclonal antibodies such as lerdelimumab, metelimumab, fresolimumab and IMC-TR1, and anti-sense mRNA molecules such as trabedersen and lucanix have yielded promising results in preclinical research and clinical trials. However, none of these compounds have yet been approved for clinical use due to the severe side effects observed in some patients, including cardiac toxicity, gastro-intestinal symptoms, fatigue, skin rash and epistaxis [33].

While much has been written on the role of TGF-β in metastasis, there is little information on the mechanisms that govern the movement of tumor cells from tissues into the lymphatic flow and towards the lymph nodes. We demonstrate that TGF-β pretreatment increases the chemotaxis, adhesion and transmigration of H157 cells, a cell line derived from squamous cell lung carcinoma, across monolayers of primary lymphatic endothelial cells of the lung. This dynamic change is accompanied by an increase in the expression of metastasis-related genes and a switch from amoeboid to mesenchymal-like cellular movement. Mesenchymal cell movement has been associated with the formation of focal adhesion contacts, a process in which integrins play a prominent role [34].

TGF-β triggers a complex network of signaling cascades that appear to involve cross-talk between integrins and TGF-β [35]. We observed an increase in the expression of several integrins at both the mRNA and protein levels that was particularly notable in the case of β3 integrin. This observation is consistent with previous reports describing TGF-β-induced increments in β3 integrin mRNA and protein expression, and αvβ3 surface expression in human lung fibroblasts via a β3 integrin, c-Src- and p38 MAPK-dependent pathway [36].

The expression of αvβ3 integrin in tumor cells has been associated with poor prognosis and increased metastasis in several carcinoma types, including osteosarcoma, pancreas and breast cancers [37-39]. In the present study, we observed decreased tumor cell adhesion and transmigration across monolayers of lymphatic endothelial cells when β3 integrin was blocked or silenced in tumor cells. Blockade of the β3 integrin ligands L1CAM and CD31 reduced tumor cell transmigration, supporting the role of active adhesion mechanisms in tumor cell transit across lymphatic endothelial cells in our experimental conditions. Indeed, previous works described binding of αvβ3 integrin as expressed by melanoma cells to blood vascular endothelium via endothelium-expressed L1CAM [40,41]. Furthermore, hypoxia has been show to induce L1CAM-mediated breast cancer cell adhesion to tumor microvasculature [42].

The role of β3 integrin in metastasis is not restricted to cell adhesion and it is also involved in the regulation of TGF-β bioavailability. In fact, the TGF-β-mediated induction of β3 integrin has been described as part of a positive feed-back loop in which β3 integrin facilitates TGF-β activation [43] by binding to the RGD domains in the complexes formed between TGF-β and the Latent Associated Peptide (LAP). This activation contributes to TGF-β-stimulated cancer metastasis in mammary epithelial cells [43]. The active cross-talk between TGF-β and integrins is triggered in tumors in response to hypoxia, oxidative stress or therapy, and it promotes tumor survival. For example, radiotherapy increases αvβ3 integrin expression as a survival mechanism in NSCLC H157 and H460 cell lines and consequently tumor growth is reduced by a combination of radiotherapy and treatment with the β3 integrin antagonist Cilengitide [44]. We observed increased survival and decreased tumor size in mice injected with β3 integrin-deficient cells as compared with those injected with β3 integrin-competent cells. Moreover, the effects of the TGF-β inhibitory peptide P144, which significantly enhances survival and attenuates tumor growth, were more dramatic in mice injected with β3-integrin-deficient cells. Treatment with P144 has been shown to inhibit tumor growth [45], angiogenesis [46] and metastasis [47], and to potentiate the efficacy of anti-tumor immunotherapy [48] in several animal tumor models.

When we analyzed lymph node affectation, we found that the inhibition of stromal TGF-β with P144 greatly diminished the appearance of tumor cells in the lymph nodes of animals injected with untreated H157 cells. These results are consistent with previous findings highlighting the role of stromal-produced TGF-β in the establishment of metastasis from primary tumors [49]. Remarkably, silencing of β3 integrin in the same tumors also reduced tumor cell transit to the lymph nodes to half the levels observed in mice injected with β3 integrin-competent cells.

Surprisingly, in vitro pretreatment of cells with TGF-β did not increase further metastasis to the lymph nodes of H157 NSCLC cells in comparison with the already high basal metastatic counts (80%) due perhaps to an excessively long end point for these experiments. In addition, TGF-β-pretreated tumor cells were resistant to separate targeting of β3 integrin silencing or stromal TGF-β inhibition with P144. This resistance may be explained by the acquisition incremented competences to bind and activate TGF-β exemplified by the increased expression of other integrins, such as αvβ5 and α4β1, and extracellular matrix degrading proteases such as MMPs.

Therefore, although the interplay between integrin β3 and TGF-β and between tumor and stromal cells in these animals remains to be fully elucidated, it is suggesting the fact that the phenotype of TGF-β1^-/-^ mice is fully reproduced in mice with mutations in the RGD binding motif in the amino acidic sequence of LAP [50]. Given the role of integrin β3 in TGF-β-mediated proteolytic activation (33) and the binding of P144 to TGF-β, we propose that these two molecules are in competition for TGF-β binding. Thus, when integrin β3 expression is low (control cells), P144 can bind more efficiently to TGF-β and exert its inhibitory activity. However, after TGF-β exposure incremented numbers of integrin β3 molecules expressed on the cell membrane bind to and activate TGF-β, thereby competing P144 binding to its target. In keeping with this hypothesis, mice injected with tumor cells that were pretreated with TGF-β but in which integrin β3 expression was silenced responded to P144 treatment with significantly impaired metastasis to the lymph nodes. These findings suggest that TGF-β pretreated cells are primed for subsequent activation by stromal TGF-β to increase their metastatic potential.

This is not the first time combined treatments that include TGF-β inhibitors have been proposed. Indeed, several studies have demonstrated that the administration of TGF-β inhibitors in combination with immune-stimulating vaccines or cytotoxic agents improve the efficacy of current TGF-β-based therapies [51]. However, in the case of integrin inhibiting peptides, caution is advised as for example, the inhibition of β1 integrin in models of mammary carcinoma activates the expression the β3 integrin and TGF-β mediated metastasis [52]. Accordingly, the correct integrin/TGF-β interaction must be identified before embarking upon complex therapeutic approaches.

## Conclusions

In this work we provide preclinical data to support the combined targeting of TGF-β and β3 integrin as a promising therapeutic approach to attenuate lung cancer metastasis to the lymph nodes in those tumors that are refractory to TGF-β directed monotherapy.

## Competing interests

The authors do not have any financial competing interest to disclosure.

## Authors’ contributions

ES: performed de in vitro experiments, treated animals, extracted data and analyzed it. SG: performed the animal experiments, histologies and video recording of the cells. JD, helped to set up the animal model and supported all the experimentation performed with P144 inhibitory peptide. XM, helped with statistics and confocal imaging. RP, contributed to all the in vitro analysis of cell migration and adhesion to different substrates. PA designed and supervised all the experiments in which anti L1-CAM antibodies were used. AR: design the project and experimental approach, supervised all the results and wrote the manuscript. All authors read and approved the final manuscript.

## Supplementary Material

Additional file 1: Figure S1 NSCLC cell lines respond to TGF-β-mediated signaling. (A) Induction of Smad2 phosphorylation 30 min after pretreatment with TGF-β in the NSCLC cell lines A549, H157 and H1299, both in the presence or absence of the TGF-β inhibitor P144 (20 or 200 ng/ml) and SB431542 for H157 cells. (B) Chemotaxis of TGF-β-treated NSCLC cells in fibronectin (10 pg/ml) or vitronectin (1 μg/ml) coated Boyden chambers (**p < 0.001, Mann–Whitney *U*-test). (C) TGF-β-induced cell migration towards the chemotactic cytokines CXCL12, CCL21 and MIP1α (**p < 0.001, Mann–Whitney *U*-test). Click here for file

Additional file 2: Figure S2 Fold changes in the expression of genes related to cell migration after TGF-β exposure of H157 NSCLC cells. (A) Fold values obtained from SABiosciences RT^2^ Profiler™ PCR Array from those genes that presented differential expression. (B) Confirmation by Real-Time PCR analyses of the differential expression of some genes in experiments performed independently. Click here for file

Additional file 3: Figure S3 Surface expression of L1CAM and CD31 on H157 cells and LECs. Flow cytometry analysis of L1CAM and CD31cell surface expression on LECs (A) and H157 cells (B) with or without prior exposure to TGF-β. LECs were also incubated with TNFα as a control. The fold-change with respect to untreated cells is indicated in the figure. Click here for file

## Supplementary experimental procedures

**Western blots** - Primary antibodies against TGF-β1 (1:300) and TGF-βRII (1:600) were purchased from R&D Systems (Minneapolis, USA), while anti-p-Smad2 (1:2000) was obtained from Chemicon and anti-Smad 2/3 (1:2000) from Cell Signaling.Migration, chemotaxis and flow cytometry were performed as described previously (23).
