# Supplementary figures and images for: Combined targeting of TGF-β1 and integrin β3 impairs lymph node metastasis in a mouse model of non-small-cell lung cancer
Source: Mol Cancer. 2014 May 19;13:112. doi: 10.1186/1476-4598-13-112 (PMC4049383; doi:10.1186/1476-4598-13-112)

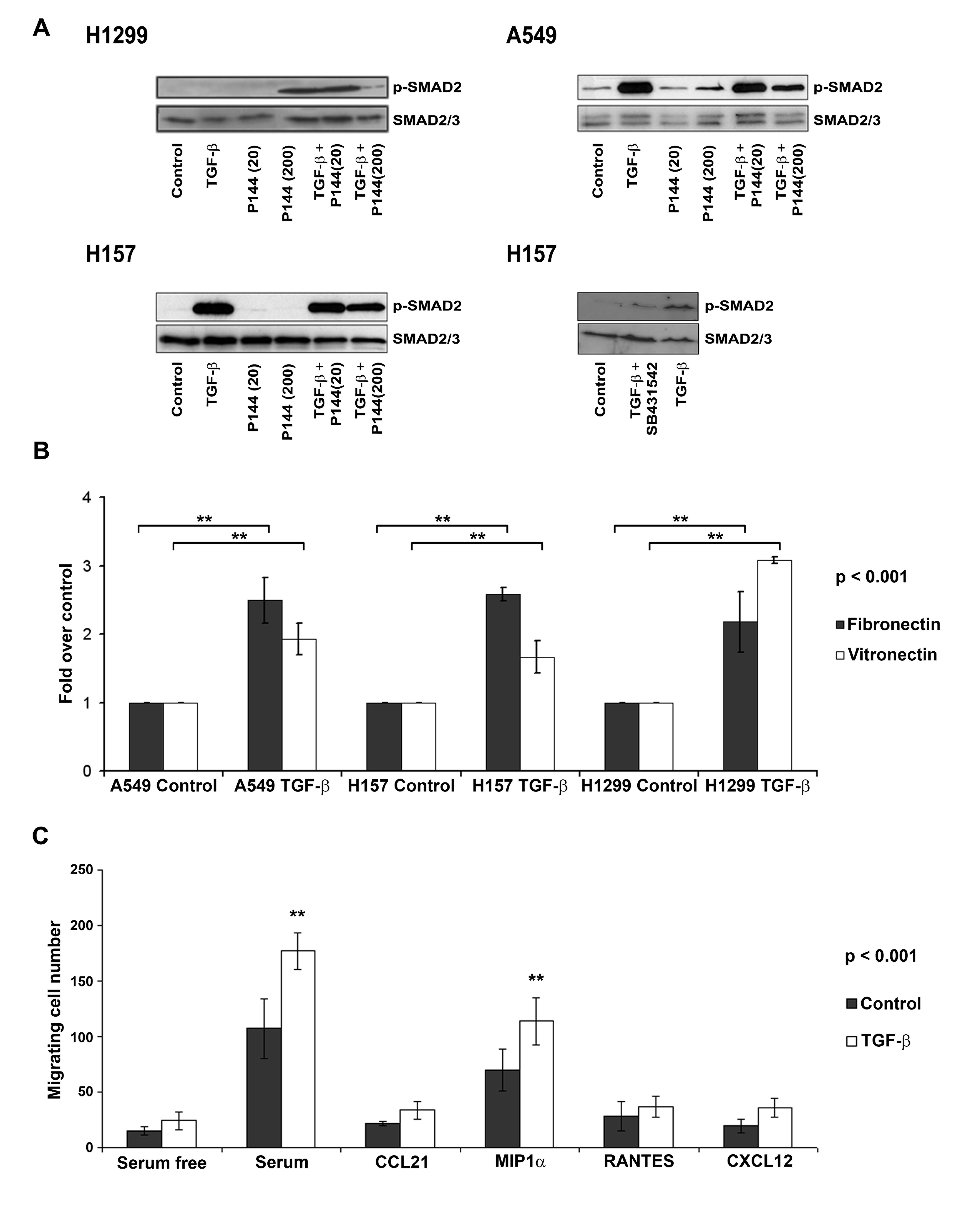

Supplement: Additional file 1: Figure S1 — NSCLC cell lines respond to TGF-β-mediated signaling. (A) Induction of Smad2 phosphorylation 30 min after pretreatment with TGF-β in the NSCLC cell lines A549, H157 and H1299, both in the presence or absence of the TGF-β inhibitor P144 (20 or 200 ng/ml) and SB431542 for H157 cells. (B) Chemotaxis of TGF-β-treated NSCLC cells in fibronectin (10 pg/ml) or vitronectin (1 μg/ml) coated Boyden chambers (**p < 0.001, Mann–Whitney U-test). (C) TGF-β-induced cell migration towards the chemotactic cytokines CXCL12, CCL21 and MIP1α (**p < 0.001, Mann–Whitney U-test). [file 1476-4598-13-112-S1.tiff]
